# Induction of Systemic Resistance in Cucumber by Hypovirulent Binucleate *Rhizoctonia* against Anthracnose Caused by *Colletotrichum orbiculare*

**DOI:** 10.21315/tlsr2019.30.1.7

**Published:** 2019-01-31

**Authors:** A. Muslim, Mitsuro Hyakumachi, Koji Kageyama, Suwandi Suwandi

**Affiliations:** 1 Department of Plant Protection, Faculty of Agriculture, Sriwijaya University, Jl. Raya Palembang-Prabumulih, Km. 32, Inderalaya, Ogan Ilir 30662, Indonesia; 2 Laboratory of Plant Disease Science, Faculty of Agriculture, Gifu University, Yanagido 1-1,501-1193 Gifu, Japan; 3 River Basin Research Center, Gifu University, Gifu 501-1193, Japan

**Keywords:** Hypovirulent Binucleate *Rhizoctonia* (HBNR), Induced Systemic Resistance, *Colletotrichum orbiculare*, *Cucumber*

## Abstract

Treatment with hypovirulent binucleate *Rhizoctonia* (HBNR) isolates induced systemic resistance against anthracnose infected by *Colletotrichum orbiculare* in cucumber, as there were no direct interaction between HBNR and *C. orbiculare*. This is because of the different distances between HBNR and *C. orbiculare*, where the root was treated with HBNR isolate and *C. orbiculare* was challenged and inoculated in leaves or first true leaves were treated with HBNR isolate and *C. orbiculare* was challenged and inoculated in second true leaves. The use of barley grain inocula and culture filtrates of HBNR significantly reduced the lesion diameter compared to the control (*p* = 0.05). The total lesion diameter reduction by applying barley grain inoculum of HBNR L2, W1, W7, and Rhv7 was 28%, 44%, 39%, and 40%, respectively. Similar results was also observed in treatment using culture filtrate, and the reduction of total lesion diameter by culture filtrate of HBNR L2, W1, W7, and Rhv7 was 45%, 46%, 42%, and 48%, respectively. When cucumber root was treated with culture filtrates of HBNR, the lignin was enhanced at the pathogen penetration, which is spread along the epidermis tissue of cucumber hypocotyls. Peroxidase activity in hypocotyls in the treated cucumber plant with culture filtrates of HBNR significantly increased before and after inoculation of pathogens as compared to the control. Significant enhancement was also observed in the fast-moving anodic peroxidase isozymes in the treated plants with culture filtrates of HBNR. The results showed the elicitor(s) contained in culture filtrates in HBNR. The lignin deposition as well as the peroxidase activity is an important step to prevent systemically immunised plants from pathogen infection.

## INTRODUCTION

Induced resistance is the phenomenon in which a plant, once appropriately stimulated, exhibits an enhanced resistance upon challenge inoculation with a pathogen. It can be localised as well as systemic, and can be induced by limited pathogen infection, virulent or avirulent pathogens, certain non-pathogenic bacteria, cell wall fragments, plant extracts and certain chemicals (Van Loon 2000; Walters 2010).

Concerns about impacts of agrichemicals on food safety and the environment are related to the danger of the synthetic pesticide utilisation, leading plant pathologists to develop another sustainable control for managing plant disease (Hyakumachi *et al*. 2014). Elicitors of host resistance are a potential alternative control to plant diseases (Lyon *et al*. 1995).

Several investigations have reported that cucumber anthracnose caused by *Colletotrichum orbiculare* could be effectively control by endophytic *Streptomyce*s (Shimizu *et al*. 2009), Rhizobacteria, *Bacillus pumilus*, *Bacillus subtilis* and *Curtobacterium flaccumfaciens* (Raupach & Kloepper 2000). Some studies show induced systemic resistance in cucumbers against antrachnose using biotic and abiotic elicitor. Meera *et al*. (1994) demonstrated that a sterile fungus and *Phoma* sp. were reliable in activating the systemic resistance of cucumber against the anthracnose disease. Koike *et al*. (2001) demonstrated that fungi isolated from zoysiagrass (*Zoysia tenuifolia*) rhizosphere (*Penicillium*, *Trichoderma*, *Phoma, Fusarium*, and a sterile fungus) significantly induced systemic resistance against cucumber anthracnoses. This is done through lignification enhancement and superoxide generation. Tian *et al*. (2008) reported that application of *Pieris rapae* extract onto the first true cucumber leaves effectively brought about systemic resistance against cucumber anthracnose with the enhancement of peroxidase and polyphenoloxidase. Lin *et al*. (2014) demonstrated that protein lysis buffer and a non-ionic detergent agent applied to separate cell membrane complexes (Nonidet P-40) is effective to weaken cucumber anthracnose by triggering genes related to disease resistance (peroxidase and pathogenis associated with protein 1-1a, acidic class III chitinase, phenylalanine ammonialyase 1). Research on hypovirulent binucleate *Rhizoctonia* (HBNR) as a potential biocontrol agent against *Fusarium* diseases in tomatoes and spinach have been recently reported in our investigations with a mechanism that might be induced resistance (Muslim *et al*. 2003a, 2003b, 2003c). There has also been an investigation of HBNR as an agent of induced systemic resistance (ISR) on beans against *Rhizoctonia solani* or *C. lindermuthianum* (Xue *et al*. 1998); they also protected cotton against alternaria leaf spot and rhizoctonia damping-off with ISR (Jabaji-Hare & Neate 2005). However, until now, there has been no report of the use of HBNR as an agent of ISR on cucumber against anthracnose pathogen *C. orbiculare* (= *C. lagenarium*).

In general, ISR in plants is clearly defined as a set of induced defense responses, including the creation of cell wall lytic enzymes. For example, 1,3-β-glucanases and chitinases (Lowton & Lamb 1987) enhance the activities of peroxidase and lignin deposition, callose, hydroxyproline-rich glycoprotein (Hammerschmidt & Kuc 1982; Hammerschmidt *et al*. 1982; Hammerschmidt *et al*. 1984); and phytoalexins (Ebel 1986).

Various agents both abiotic and biotic inducer (e.g., virulent or avirulent pathogens, nonpathogen microorganisms, cell wall fragments, plant extracts, and synthetic chemicals) have been documented to induce resistance after challenging with pathogen attack, both locally and systemically (Walters *et al*. 2005). Plants possess various inducible defense mechanisms to protect themselves against pathogens. These defense mechanisms include preexisting physical and chemical barriers, as well as inducible defense responses. The pre-existing biochemical defense mechanisms include phenolics, phenolic glycosides, unsaturated lactones, saponins, cyanogenic glycosides, glucosinolates, 5-alkylated resorcinols and dienes (Osbourn 1996). The inducible defenses include the production of reactive oxygen species (ROS), hypersensitive response, reinforcement of cell wall, phytoalexins production and pathogenesis-related (PR) proteins (Mellersh & Heath 2004).

This study aims to investigate HBNR capacity in inducing systemic resistance in cucumber against *C. orbiculare*. The study was designed to reveal if induced resistance in cucumber is correlated with enhanced systemic lignification and peroxidase activity.

## MATERIALS AND METHODS

### Isolates

Hypovirulent binucleate *Rhizoctonia* isolate of W1, W7 (AG-A), L1 (AG-Ba), and Rhv7 (unknown anastomosis group) obtained from soil samples were used as biocontrol agents. The pathogens used in this study were *Colletotrichum orbiculare* (Berk & Mont.) Arx (=*Colletotrichum lagenarium* (Pass.) Ellis & Halst.) isolate 104T, which were obtained from infected cucumber plants.

### Plants

Throughout the experiment, cucumber cv. Gibai was used. Before the sowing, seeds were sterilised with 70% ethyl alcohol for one minute, and 1% of NaOCl for 20 min. Finally, they were rinsed in sterilised distilled water three times.

### Inoculum Preparation

Isolates of pathogen *C. orbiculare* were cultured on potato dextrose agar (PDA) as long as seven days without exposure to light. The temperature was maintained at 25°C. A sterilised glass bar from the cultures with added sterile water, and scraped the spore suspensions. The spore suspension was then filtered through eight layers of sterile gauze. The isolates were set as two inoculum forms: barley grain inoculum and culture filtrate.

The following procedure was used for preparation of barley grain inoculum: Each isolate was cultured in PDA for three days without light and at room temperature. Five 5 mm mycelial disks of the culture were applied to 100 g of moist autoclaved barley grains (1:1, w/v dry barley grains/distilled water) collected in a 500 mL Erlenmeyer flask. The cultures were maintained and regularly shaken for 10 days at 25°C to produce well-colonised inoculum with HBNR. The inoculum was naturally dried for around 10 days. They were then kept refrigerated at 4°C until use.

The following procedure was used for the culture filtrate (CF): Two mycelial disks of each HBNR isolate obtained from the culture growing on PDA were put into a 20 mL flask with 50 mL of potato dextrose broth (pH 6.5). The isolates were grown in static conditions at 23°C–25°C for 10 days without light. The CF separated from the mycelia. Next, the CF was filtered three times over three layers of Whatman filter paper number 2. The CF was also filtered and sterilised using millipore filtration (0.45 μm Millipore filters, Millipore Products Division, Bedford, USA).

### Cucumber ISR Assays

#### Experiments with barley grain inocula

Each sterilised plastic pot, sized ø 6 cm × 7.5 cm, was filled with the colonised barley grain inocula mixture (2%, w/w) with as much as 120 g of potting medium. The previously-sterilised (with 0.5% NaOCl) cucumber seeds were added to the mixture. Each pot was given one seed. Next, the plants were cultivated at 25°C. This required 21 days in a growth chamber with a 14 h light (24,000 lux) per dark period. The plants that were grown in the potting medium with untreated barley grain inocula were used as a control. Each inocula of HBNR isolate was inoculated on six plants as replication and the experiment was repeated twice.

#### Experiment with culture filtrates (CF)

The plastic pots (autoclavable, ø 6 cm × 7.5 cm) containing about 120 g of potting medium were heated in autoclaves. The surface-sterilised cucumber seeds were sown in each pot. The plants were maintained in a similar manner as previously described. The first true leaves of 21-day-old cucumber plants were soaked with CF for 1 min. The plants were inoculated after 24 h of incubation. Each CF of HBNR isolate was applied on six plants as replication and the experiment was repeated twice.

#### Challenge inoculation

The second true leaves were inoculated with 20 individual drops (each drop was 10 μl) of spore suspension of *C. orbiculare* (5 × 10^5^ spores/mL). The disk of lens paper (ø 5 mm) was covered on every drop toward the run-off prevention. This was done to ensure the distribution of equal numbers of spores along the leaf surfaces. The inoculated plants were maintained for 48 h without light at 25°C in a humid chamber (85%–90% RH). After that, for six days the inoculated plants were brought to the growth chamber. The total number per leaf and diameter of lesion per inoculated drop were measured.

### Testing for Lignin Formation

The cucumber seeds were grown on damp sterilised filter paper. Next, they were incubated for a week without light at 25°C. The roots of the seedlings were put in 5.0 mL of CF and incubated for one day. Then, with 10 μl drops of spore suspension (5 × 10^5^ spores/mL) of *C. orbiculare*, the hypocotyls of the treated seedlings were inoculated. Next, the inoculated seedlings were incubated for 20 h. The epidermal strips of the seedling hypocotyls were stained with toluidine blue O or phloroglucinol-HCL (Sherwood & Vance 1976). They were observed under the microscope to reveal percentage of lignification.

Spores of *C. orbiculare* germinated 90% or more on cucumber hypocotyls. The degree of lignin deposition was evaluated by determining the percentage of germinated spores together with appressoria around which lignin depositions were induced. For each treatment 100 germinated spores were evaluated.

### Protein Extraction and Determination

Treated cucumber root seedlings with CF of HBNR and challenge inoculated with *C. orbiculare* were prepared as described previously in section of testing for lignin formation. Samples were collected from seedlings prior to the time of challenge inoculation and again 8–48 h after the challenge inoculation. All samples were immediately frozen at −80°C until peroxidase assays were performed. Only the hypocotyls of the cucumber seedlings were used for protein extraction. These samples were homogenised in 5 mL of 0.05 M sodium phosphate buffer at pH 6.0 per 1 g sample with a cold mortar and pestle. The extract was centrifuged at 10,000 rpm for 10 min at 4°C, and the supernatant was used to analyse the peroxidase activity. To determine the protein contents of these extracts, the Lowry method (Lowry *et al*. 1951) was used with bovine serum albumin as the standard.

### Assay for Peroxidase Activity

Peroxidase activities were assessed following the method of Dalisay and Kuc (1995). They were determined using guaiacol, which acted as the hydrogen donor. The reaction mixture (3 mL) contained 0.25% (v/v) guaiacol in 1 mM sodium phosphate buffer at pH 6.0 with 100 mM hydrogen peroxidase. In order to catalyse the reaction, one-tenth ml crude enzyme extract was added and continued with colorimetrically at 470 nm min^−1^ mg^−1^ protein.

### Detection of Peroxidase Isozymes by Gel Electrophoresis

Native PAGE was done with a PhastSystem (Pharmacia LKB, UK). Extracts were adjusted to the same protein concentration with phosphate buffer and then loaded onto an 8%–25% gradient gel. A peroxidase isoenzyme was made visible by immersing the extracts in gels of 1% *o*-dianisidine solution. After 10 min, the gels were cleaned with distilled water. They were then placed into 0.06% H_2_O_2_ solution to concretely show the peroxidase isoenzyme bands.

### Data Analysis

The experiments in this study were designed in completely randomised designs. Total lesion numbers, anthracnose lesion diameters, and lignin formation in this study were compared using Fisher’s least significant difference (LSD) test at *P* = 0.05 and *P* = 0.01.

## RESULTS

### ISR in Cucumber Against Anthracnose with HBNR

This study showed that the use of barley grain inoculum and CF of HBNR isolates significantly (*P* = 0.05) decreased total anthracnose lesion diameter compared to the control (Table 1). However, no significant reduction was observed in total lesion number (Table 1). The reduction of total lesion diameter by barley grain inoculum of HBNR L2, W1, W7, and Rhv7 was 28%, 44%, 39% and 40%, respectively. Similar results were also observed in the treatment with CF; the reduction of total lesion diameter by CF of HBNR L2, W1, W7, and Rhv7 was 45%, 46%, 42%, and 48%, respectively (Table 1).

### Lignin Formation and Peroxidase Activities in Cucumber Hypocotyls Treated with HBNR

**Figure 1:**
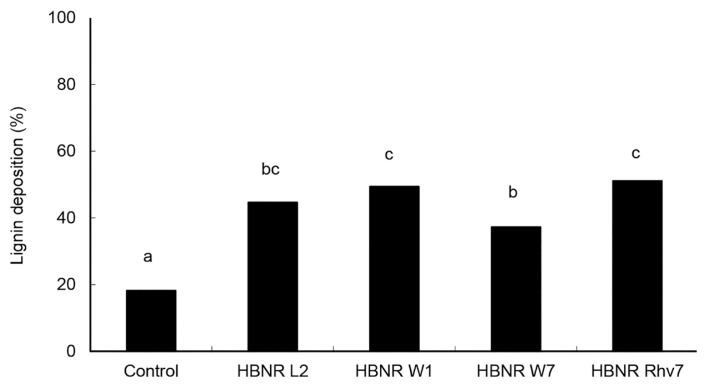
Lignification of hypocotyls of cucumber seedlings induced by culture filtrates of HBNR, following challenge inoculation with *Colletotrichum orbiculare*. Cucumber seedlings treated with sterilised distilled water were used as control. The hypocotyls of treated plants were challenged with 5 μl drops of 10^5^ spores/ml of *C. orbiculare* at 10 locations. Bars labeled with the same letter are not significantly different according to Fisher’s least significant difference test (*P* = 0.01).

Lignin formation was observed as the intense blue and green colours of the lignified cell walls. Cucumber hypocotyls pretreated with CF of HBNR L2, W1, W7, and Rhv7 significantly increased lignin deposition in places that had been infected by *C. orbiculare* compared to the control treatment (Fig. 1). Cucumber seedlings treated with CF of HBNR L2, W1, W7, and Rhv7 increased lignin deposition by 1.45-fold, 1.71-fold, 1.04-fold, and 1.81-fold, respectively, relative to control.

**Figure 2:**
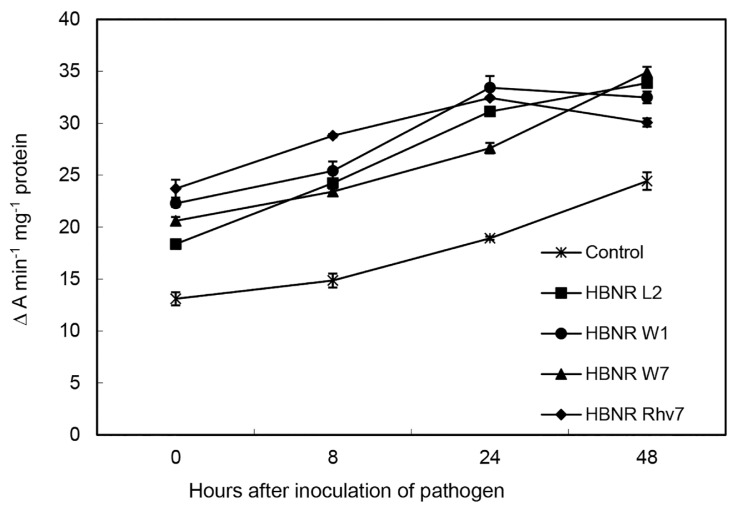
Time course of peroxidase activity in hypocotyls of cucumber after treating the root with HBNR isolates and challenge inoculating with *C. orbiculare*. Peroxidase activity is expressed as changes in absorbance min^−1^ mg^−1^ protein. Cucumber seedlings treated with sterilised distilled water were used as control. Data are the mean of three replications with five seedlings (cucumber) per replication. Bars represent standard error of the mean. 0 h indicates time before pathogen inoculation.

Peroxidase activities in cucumber hypocotyls sampled at varying times before and after challenge inoculation were higher in the plant treated with HBNR compared to the control (Fig. 2). Treatment with HBNR L2, W1, W7 and Rhv7 increased peroxidase activities by 40%, 70%, 57%, and 81%, respectively, before inoculation of *C. orbiculare*, and by 39%–64%, 33%–94%, 43%–58%, and 23%–94%, respectively, relative to control after inoculation of *C. orbiculare*.

**Figure 3:**
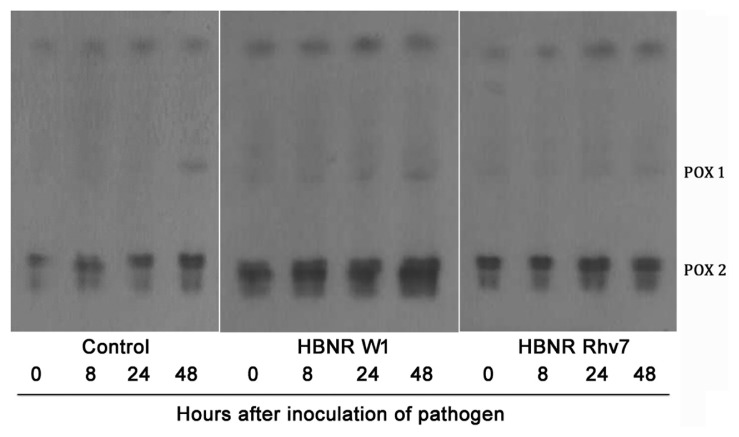
Electrophoresis patterns of peroxidase isozyme (POX 1 and POX 2) cucumber seedlings treated with HBNR (Protein concentration was 0.1 mg/ml). Cucumber seedlings treated with sterilised distilled water were used as control.

Two peroxidase isozymes (isoforms 1 and 2) were found in cucumber hypocotyls. The fast-moving anodic peroxidase isozymes were enhanced gradually after challenge inoculation. The peroxidase activities increased in the isoform 2 in the seedlings treated with HBNR compared to the control, at all sampling times, according to band intensity and width (Fig. 3). Isozyme type 1 had a minor activity band and was observed after 48 h of pathogen inoculation either on inoculated or non-inoculated with pathogen.

## DISCUSSION

This study reveals that treatment with HBNR isolates suppresses disease development of anthracnose in cucumber. The disease development suppression seemingly resulted from plant’s ISR, as separated inoculation sites between HBNR and *C. orbiculare*, where the root was employed with HBNR isolates, and *C. orbiculare* was inoculated on the leaves, or the first true leaves were treated with HBNR isolates and *C. orbiculare* was challenge inoculated on the second true leaves. Thus, HBNR and pathogen application sites were separated spatially, and no HBNR isolates could be recovered from the second true leaves. The result of this research supports the evidence that the mechanism of protection from *R. solani* by HBNR is induced resistance (Cardoso & Echandi 1987; Poromarto *et al*. 1998).

A report presented by Xue *et al*. (1998) showed that inoculation of bean hypocotyls with HBNR induced systemic resistance and protection of the roots and cotyledon to later challenges not only with the root rot pathogen *R. solani* but also with the anthracnose pathogen *C. lindemuthianum*. This study applied HBNR as barley grain inoculum, and CF induced systemic resistance in cucumber plants against *C. orbiculare*. Similar methods were used by Meera *et al*. (1994) and Koike *et al*. (2001), in which plant-growth-promoting fungi (PGPF) were applied at the root as barley grain inoculum, mycelia inoculum, or culture filtrates. This induced systemic resistance in cucumber after being challenged with *C. orbiculare* in leaves. Another study reported that germinating tomato seeds for one week in chemicals of b-aminobutyric acid (BABA) and jasmonic acid (JA) solutions promoted seed germination efficiency and induced resistance in four-week-old plants (Luna *et al*. 2016).

In this study, when HBNR CF was applied at the cucumber roots, lignin was enhanced at the attempted penetration by the pathogen in the epidermal tissues of cucumber hypocotyls. Enhanced lignin deposition was positively correlated with significant reduce of lesion development. Lignin may improve plant resistance against fungal infection through enhanced physical barrier and chemical direct toxicity through their toxic derivatives such as phenolic compounds (Xue & Yi 2017). Our results also show that peroxidase activity in hypocotyls in the treated cucumber plant with HBNR significantly increased before and after inoculation of the pathogen compared to the control. Significant enhancements were also observed in the fast-moving anodic peroxidase isozymes (isoform 2) in the plants treated with HBNR. Isoform 1 may have less significant role in induce resistance since it showed a minor activity and found on both inoculated and non-inoculated hypocotyl. This supports the finding by Xue *et al*. (1998) that inoculation of bean hypocotyls with HBNR induced systemic resistance, and this was positively correlated with peroxidase. Arora and Bajaj (1985) and Krstic *et al*. (1997) also reported that infection of mung bean and strawberry with binucleate *Rhizoctonia* resulted in an increase in peroxidase activity. Peng *et al*. (2004) found that pretreated cucumber seedlings with pectinase extract derived from *Penicillium oxalicum* BZH-2002 fermentation products resulted in induced resistance toward cucumber scab *Cladosporium cucumerinum* through the increased defense-related enzymes, polyphenol oxidase, and peroxidase.

Increased peroxidase activity is also well observed in rhizobacteria-induced systemic resistance. Chandrasekaran and Chun (2016) demonstrated that treating tomato plants with *Bacillus subtilis* CBR05 significantly enhanced the activities of antioxidant enzymes including peroxidase. Yanti (2015) reported that rhizobacteria enhanced peroxidase enzyme activity. The isolate PK2Rp3 (*Serratia marcescens* strain N2.4) showed the highest activity of both roots and leaves of 0.058 μg · mL^−1^ and 0.053 μg · mL^−1^.

According to Dean and Kuc (1987) and Hammerschmidt *et al*. (1984), lignin deposition was considered a crucial phase of pathogen suppression in systemically immunised plants. Vance *et al*. (1980) reported that rapid lignin deposition might lead to the production of chemical or physical barriers to pathogen infection. Furthermore, peroxidases accelerate the ending polymerization step of lignin synthesis, resulting in the enhanced capability of protected tissue (Gross 1979). In the other studies, the enhanced peroxidase activities are often related to resistance phenomenon such as the production of lignin (Hammerschmidt & Kuc 1982; Ride 1975). Peng and Kuc (1992) discovered the implications of peroxidase towards oxidative defense mechanisms in treated plants with infections. The peroxidase-generated hydrogen peroxidase directly functions as an antimicrobial agent. In our study, when plant treated with barley grain inoculum and CF of HBNR isolates, significant reduction was observed in total lesion diameter. However, no significant reduction was observed in total lesion number. This result indicated that increased lignification and peroxidase activities observed in this study did not restrict total penetration of *C. orbiculare*. The data suggest that the involvement of other defense mechanism(s) acting at the level of restricting lesion development to fungal infection. Lignification and peroxidase activities, alone or collectively, are not sole determinants for induced systemic resistance. Van Loon (2000) indicated that induced resistance is the result of multi-mechanisms. Therefore, it is necessary to investigate further other mechanisms, alone or collectively, involved in systemic resistance against *C. orbiculare*. Further research is needed to identify other PR-proteins that may be involved in the mechanism of cucumber ISR from HBNR.

The abilities of HBNR isolates to induce systemic resistance in cucumber against anthracnose and to enhance lignin deposition and peroxidase activity as well as their effectiveness against *Fusarium* diseases in tomato and spinach in our previous studies (Muslim *et al*. 2003a, 2003b, 2003c), shows significant potential as a bio-control agent to manage *Colletotrichum orbiculare* and other diseases.

## CONCLUSION

Plant treated with barley grain inoculum and culture filtrate of HBNR isolates effectively reduced lesion development of anthracnose in cucumber with induced systemic resistance mechanism through enhancing lignin deposition and peroxidase activity.

## Figures and Tables

**Table 1:** Effect of HBNR isolates on the total lesion number and lesion diameter on leaves of cucumber plants that have been challenge inoculated with *Colletotrichum orbiculare*.

Treatments	Total lesion number^a^		Total lesion diameter (mm)^a^	
	
	BGI^b^	CF^c^	BGI	CF
Pathogen	19.5 a	16.8 a	123.5 b	89.3 b
HBNR L2	16.8 a	12.5 a	89.5 a	48.7 a
HBNR W1	16.0 a	13.1 a	68.7 a	48.1 a
HBNR W7	17.2 a	12.9 a	75.5 a	51.8 a
HBNR Rhv7	16.8 a	11.7 a	74.5 a	46.5 a

Notes:

a Mean of two trials each with six plants per treatment. Values followed by the same letter do not differ significantly (*P* = 0.05) according to Fisher’s least significant difference test.

b Plants were grown in potting medium amended with barley grain inoculum (BGI) of HBNR isolates (1%, w/w) for 21 days and challenge inoculated with 10 μl drops of 5 × 10^5^ spores/ml of *C. orbiculare* at 20 locations on the second true leaves.

c The first true leaves of 21-day-old cucumber plants grown in potting medium were treated with CF of HBNR and challenge inoculated with *C. orbiculare* on the second true leaves as described above.
